# Effect of cyclic loading on the stability of screws placed in the locking plates used to bridge segmental bone defects

**DOI:** 10.1002/jor.24838

**Published:** 2020-09-09

**Authors:** Remigiusz M. Grzeskowiak, Laura R. Freeman, David P. Harper, David E. Anderson, Pierre‐Yves Mulon

**Affiliations:** ^1^ Large Animal Clinical Sciences University of Tennessee College of Veterinary Medicine Knoxville Tennessee USA; ^2^ The Center for Renewable Carbon, Institute of Agriculture University of Tennessee Knoxville Tennessee USA

**Keywords:** fracture, implant stability, locking plates, locking screws, orthopedics, peak reverse torque, segmental defect

## Abstract

The objective of this study was to evaluate the ex vivo effect of cyclic loading on the stability of screws placed in locking plates used to bridge segmental bone defects. The primary interface stability was assessed using peak reverse torque. Eighteen, 8‐hole stainless‐steel 4.5 mm locking plates and 4.0‐mm self‐tapping locking‐head screws were used to stabilize 40‐mm segmental defects in goat tibiae. Treatment groups included control constructs without cyclic loading (*n* = 6) and constructs tested to 5000 (*n* = 6) and 10,000 cycles (*n* = 6) of 600 N compressive axial loading. The insertion of all screws was standardized to 400 N‐cm insertion torque. Peak reverse torque was measured immediately after screw placement (control), or after the completion of the respective loading cycles. The difference between treatment groups was compared using univariate analysis of variance. The analysis revealed a significant difference in peak reverse torque of the screws among the treatment groups (*p* = .000). The mean reverse torque values equaled 343.5 ± 18.3 N‐cm for non‐cycled controls, 303.3 ± 25.9 and 296.0 ± 42.9 N‐cm after 5000 and 10,000 cycles, respectively. Among all treatment groups, screws placed in the distal bone segment tended to have lesser peak reverse torque reduction than those placed in the proximal segment and the difference was proportional to the number of cycles (*p* = .562; *p* = .255; *p* = .013 in control, and after 5000 and 10,000 cycles, respectively). Cyclic loading may have a negative effect on the primary stability of screws placed in locking plate constructs used to bridge segmental bone defects and could contribute to the risk of screw loosening.

## 1 INTRODUCTION

Locking plates are increasingly used to stabilize fractures and are estimated to be used in 5%–25% of all fractures repaired with osteosynthesis.^1^ The plates are particularly advantageous in osteoporotic bone with thin cortices which do not allow for the desired screw purchase required for conventional plating.^1^ Locking plates also have found application in extensive comminuted fractures in diaphyseal and metaphyseal bone regions to bridge non‐load‐sharing areas, in fractures involving prosthetic stems, as well as in periarticular fractures to provide angular stability to a joint surface.^1^ There are several indications for locking plating within specific anatomic locations, such as tibiae, including complex proximal tibial fractures (Schatzker IV), as well as tibial pilon fractures extending into diaphyseal segments.^1^, ^2^, ^3^

Locking plates enhance the screw‐plate‐bone construct stability by creating a single‐beam construct.^4^, ^5^ The term single‐beam describes a construct with no motion between the fragments of the beam, such as plate, screw, and bone.^4^, ^5^ Single beam constructs have been found to be four times stronger than load‐sharing beam constructs.^4^ In the angle‐stable fixation granted by the locking plates, shear stress created during loading is directly converted into compression forces applied to the screws and the overall construct fixation strength equals the sum of all holding strengths of the screw‐bone interfaces.^4^, ^5^, ^6^, ^7^, ^8^ The stability of each screw is therefore vital for the stability of the entire single‐beam construct.

Stability of the screw implants has been defined with primary and secondary stability.^9^, ^10^, ^11^, ^12^ Primary stability is the mechanical stability obtained by the implant immediately after its placement within the plate construct.^9^, ^10^, ^11^, ^12^, ^13^ Secondary stability is obtained by the implant throughout the osseointegration process and is directly influenced by the primary stability.^14^, ^15^ Excessive micromotion between the implant and bone (above 150 μm), and poor quality of surrounding bone have been found to significantly impair osseointegration of the implant.^16^ Peak reverse torque (PRT) is one method to assess the stability of screw implants.^10^, ^11^, ^12^, ^17^, ^18^, ^19^, ^20^ This method has been frequently employed in orthodontics^10^, ^11^, ^12^ as well as more recently in orthopedics.^18^, ^19^, ^20^ PRT measures the torque required to remove the screw implant, and therefore directly evaluates the strength of locking screw and plate interface.^10^ Screws with greater interface stability will require greater removal torque. In spite of many advantages of this method and its validation in orthodontic as well as orthopedic research, studies using PRT to evaluate the stability of screws placed in locking plate constructs are lacking.

The objective of this study was to evaluate the effect of cyclic axial compressive loading on the stability of the locking‐head screws used in locking plates to stabilize non‐load‐sharing fractures. In order to mimic the clinical scenario in which the bone segments would have little to no influence on stability, a complete segmental mid‐diaphysis tibia defect was created and stabilized with a locking plate and screw construct and then subjected to up to 10,000 cycles of compressive axial loading. The stability of locking‐head screws was evaluated measuring the PRT required to remove the screws. This study hypothesized that there will be an effect of the cyclic loading on the stability of the locking‐head screws placed in locking plate constructs.

## 2 METHODS

### 2.1 Specimen preparation

**Figure 1:**
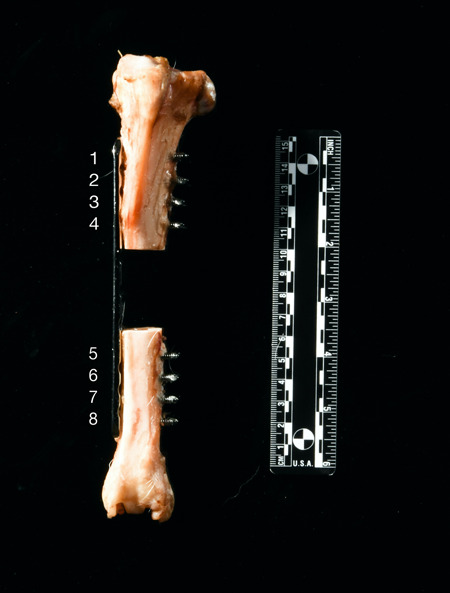
The locking plate construct before potting in polymethyl methacrylate (PMMA). The 40 mm full‐thickness segmental tibial defect was stabilized with 8 whole 140 mm long 4.5 mm locking plate. Four proximal locking‐head screws were placed in the proximal bone segment in positions from 1 to 4 and four distal locking‐head screws were placed in the distal bone segment in positions from 5 to 8 [Color figure can be viewed at wileyonlinelibrary.com]

This study population included 18 tibiae harvested from healthy, adult goats (>2 years, weight 55.52 ± 7.1 kg) having been used in a previous orthopedic study. These 18 tibiae were randomly and equally assigned to one of three treatment groups, including (1) control constructs with no cyclic loading (C; *n* = 6), (2) constructs subjected to 5000 loading cycles (G5K; *n* = 6), and (3) constructs subjected to 10,000 loading cycles (G10K; *n* = 6). The bones were removed at the level of femorotibial and tarsocrural joint and dissected from surrounding soft tissues, including ligaments and tendons. The proximal and distal end of the bones were potted in polymethyl methacrylate (Jorgensen Laboratories) within polyvinyl chloride (PVC) (Lowe's Home Improvement) molds to achieve an external diameter of 50.8 mm (Figure 1).

### 2.2 Constructs preparation

One hundred and forty milimeters long and 4.5 mm thick, 8‐hole bridging locking plates (316 Stainless Steel Orthopedic Locking Plates, Veterinary Orthopedic Implants) with a 60 mm long solid central portion between the screw holes were used to bridge a 40 mm long, segmental tibial defect. The plates were applied to the cranial, medial surface of the tibiae and fixed with eight 4.0 mm self‐tapping locking‐head screws (316 Stainless‐steel Locking‐head Screws, Veterinary Orthopedic Implants), four proximal to the segmental defect (position from 1 to 4) and four distal to it (position from 5 to 8) (Figure 1).

The first two screws were placed in position #1 (most proximal) and #8 (most distal) to fix the plate against the bone surface. For each screw position, screw holes were drilled using a 3.2 mm drill guide (Veterinary Orthopedic Implants) and an orthopedic power drill (model number: ND‐1001) with a 3.2 mm drill bit (Veterinary Orthopedic Implants). Drilling was continued until the tip of the drill bit reached beyond the far cortex. A depth gage (Veterinary Orthopedic Implants) was used to measure the depth of drilled screw holes to estimate the required length for the screws (range of length: 28–34 mm). The screws were placed manually with a hand‐held screwdriver (STAR 4.0 screwdriver, Veterinary Orthopedic Implants), but not tightened. In order to mimic a non‐load sharing fracture, a 40 mm full‐thickness osteotomy was created in the center of the tibiae with a diamond crusted bone saw (110 v Pathology Bone Band Saw, IMEB). During an osteotomy, care was taken to leave equal 10 mm distances between the screw holes adjacent to osteotomy and the margins of the osteotomy (Figure 1).

Following osteotomy, screws in positions #4 and #5 were placed as described above to fully stabilize the bone fragments. Thereafter, the remaining four screws were identically placed (position #2, 3, 6, and 7) in chronological order. All screws were placed within the plates according to Arbeitsgemeinschaft für Osteosynthesefragen/Association for the Study of Internal Fixation guidelines. During screw placement, care was taken that the cutting flute of each self‐tapping screw extended beyond the far cortex to allow for sufficient bone purchase within the far cortex. All screws extended two to three threads beyond the far cortex to ensure full engagement of the screws. After all screws had been placed, the screws were tightened using a digital hand‐held torque measuring device (Cedar DID‐4 Digital Torque Screwdriver, Sugisaki Meter CO) with the insertion torque limited to 400 N‐cm as recommended for 4.0 mm locking screws by the manufacturer and previous reports.^19^, ^21^, ^22^ Peak insertion torque (PIT) was recorded in the computer software (Microsoft Office Professional Plus 2016, Microsoft) for all placed screws.

### 2.3 Construct biomechanical testing

The constructs assigned to the control treatment group (C; *n* = 6) did not undergo biomechanical testing and PRT was measured immediately after all screws had been tightened. The results of the control PRT measurement served further as a reference point for the PRT measurements following cyclic loading.

**Figure 2:**
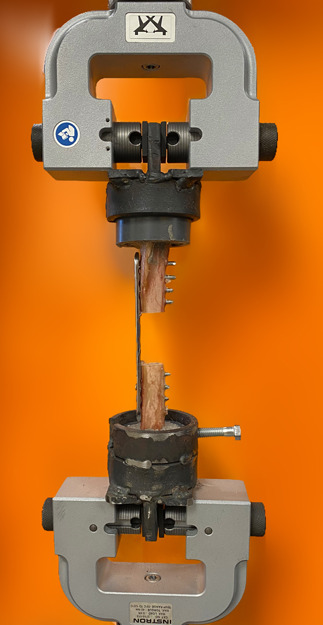
The locking plate construct within the testing frame. The specimen was placed within the custom‐made steel grips which allowed for a stable fixation within the mechanical testing frame. The screw in the bottom grip was tightened before the construct testing to prevent the rotation of the specimen during testing [Color figure can be viewed at wileyonlinelibrary.com]

In order to mimic short‐term postoperative loading conditions, constructs assigned to remaining treatment groups were subjected to either 5000 (G5K; *n* = 6) or 10,000 cycles (G10K; *n* = 6) of compressive axial loading.^23^ The constructs were mounted within a customized frame for the electromechanical testing machine (Instron 5567) and secured by custom‐made bone grips (Figure 2). The grips were made from steel with a diameter marginally exceeding the outer diameter of PVC pipes to allow for a low‐resistance specimen placement. The rate of loading was controlled throughout the experiment.

Each specimen was preloaded with 10 N compression and the compressive axial load was limited to 600 N. A load of 600 N was consistent with a compressive load applied under physiologic conditions by the average weight goat (52 ± 7.1 kg) to the tibia during walking.^24^ The loading cycles were applied to specimens with a frequency of 1 Hz. The actuator displacement as well as stiffness of each specimen was measured and recorded in real‐time by the manufacturer software (Bluehill 3). Displacement of the actuator was defined as the distance from the actuator original position to its position during the application of 600 N load.^25^, ^26^ Stiffness of the specimen was defined as the resistance to deformation during cyclic loading and described with load over the displacement curve.^25^, ^26^ After mechanical testing, PRT of each screw was measured prior to screw extraction with the hand‐held torque measuring device. The result of each measurement was recorded by the manufacturer's software (Microsoft Office Professional Plus 2016, Microsoft).

### 2.4 Statistical analysis

The measurements of PIT and PRT as well as results of biomechanical testing were recorded and organized using Excel spreadsheets (Microsoft Office Professional Plus 2016). The statistical analysis was conducted using SPSS statistics software (v.26; IBM) and the power of the study was calculated using PS power and sample size analysis software (ver 3.0, 2009). Descriptive statistics, including mean values and standard deviations, were calculated for each variable. Data in each variable was assessed for the normality of distribution using the Kolmogorov–Smirnov test and for the equality of variance using Levene's test.

Mean PIT and PRT data from all screw positions and mean PIT and PRT data from the individual screw positions (from 1 to 8) were compared between the treatment groups. Mean PIT and PRT values were further compared between the individual screw positions within the treatment groups. Results of PIT and PRT measurements were subsequently combined for the proximal (from 1 to 4) and distal (from 5 to 8) screw positions and compared within the treatment groups. The comparison was conducted using a univariate analysis of variance test and Tukey's post hoc with PIT and PRT as the dependent variables and treatment group as well as screw position as fixed effects. Further comparison of the combined screw positions was conducted using independent samples two‐tailed student *t* test.

The results of biomechanical testing, including actuator displacement and stiffness of the constructs, were compared between the treatment groups subjected to cyclic loading. The comparison was conducted using independent samples two‐tailed student *t* test. Statistical significance was established at *p* < .05. Accounting for the difference in PRT results between the treatment groups found in this study and methods of statistical analysis, the power of the study to detect the true difference was calculated to be *β* = 0.85.

## 3 RESULTS

**Figure 3:**
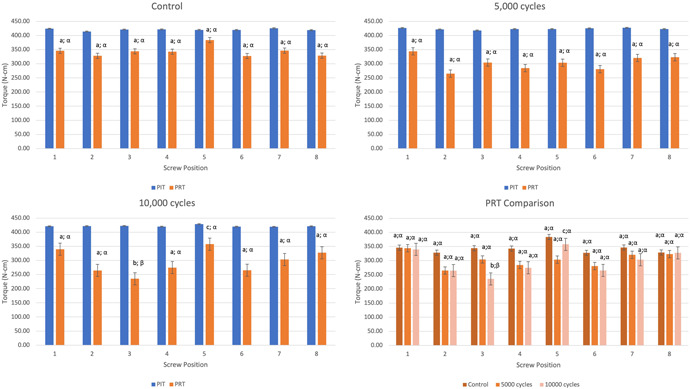
Peak insertion torque (PIT) and peak reverse torque (PRT) comparison between the treatment groups. Statistically significant difference in PRT between the screw positions within the same treatment groups are labeled using different alphabetic letters and statistically significant difference between the treatment groups for the same screw position are labeled with different Greek letters [Color figure can be viewed at wileyonlinelibrary.com]

The study revealed a statistically significant reduction in PRT of the screws placed in the locking plate construct after compressive axial loading (*p* = .000). The average measured PRT equaled 343.47 ± 18.3, 303.26 ± 25.9, and 296.04 ± 42.9 N‐cm in C, G5K, and G10K treatment group, respectively (Table 1). A significant difference in PRT was found between C and G5K treatment group (*p* = .010) as well as between C and G10K treatment group (*p* = .000) (Figure 3).

The difference in PRT between G5K and G10K was not statistically significant (*p* = .797).

**Table 1:** Mean values and SD of PIT and PRT values (N‐cm) for individual screw positions (1–8)

**Screw position**	**Control**		**5000 Cycles**		**10,000 Cycles**	
	**PIT, N‐cm**	**PRT, N‐cm**	**PIT, N‐cm**	**PRT, N‐cm**	**PIT, N‐cm**	**PRT, N‐cm**
1	424.4 ± 11.6	346.1 ± 58.7^a,α^	427.1 ± 10.8	344.3 ± 35.5^a,α^	421.4 ± 7.1	339.9 ± 23.2^a,α^
2	414.4 ± 3.6	328.0 ± 53.9^a,α^	422.0 ± 6.8	265.1 ± 62.4^a,α^	421.6 ± 13.4	264.7 ± 72.1^a,α^
3	421.2 ± 6.6	344.2 ± 48.7^a,α^	418.2 ± 8.8	304.2 ± 34.1^a,α^	422.4 ± 11.8	235.2 ± 86.7^b,β^
4	422.0 ± 4.0	342.6 ± 37.9^a,α^	423.1 ± 9.5	284.5 ± 55.8^a,α^	419.9 ± 13.3	274.8 ± 54.9^a,α^
5	420.2 ± 7.7	383.8 ± 43.3^a,α^	423.2 ± 6.6	303.4 ± 79.5^a,α^	429.1 ± 9.2	357.9 ± 64.2^c,α^
6	419.8 ± 9.9	327.7 ± 49.1^a,α^	425.5 ± 17.9	280.6 ± 56.6^a,α^	420.0 ± 9.2	265.2 ± 56.2^a,α^
7	425.9 ± 11.9	346.5 ± 64.5^a,α^	427.5 ± 12.1	320.9 ± 47.8^a,α^	419.3 ± 13.0	303.4 ± 62.9^a,α^
8	419.1 ± 5.7	328.9 ± 22.8^a,α^	422.9 ± 9.5	323.1 ± 52.7^a,α^	421.1 ± 14.0	327.4 ± 47.2^a,α^
Average	420.9 ± 3.5	343.5 ± 18.3^α^	423.7 ± 3.01	303.3 ± 25.9^β^	421.9 ± 3.1	296.0 ± 42.9^β^

*Note*: a, Statistically significant difference between the screw positions within the same treatment groups were labeled using different alphabetic letters and α, statistically significant difference between the treatment groups for the same screw position were labeled with different Greek letters.

Abbreviations: PIT, peak insertion torque; PRT, peak reverse torque.

**Figure 4:**
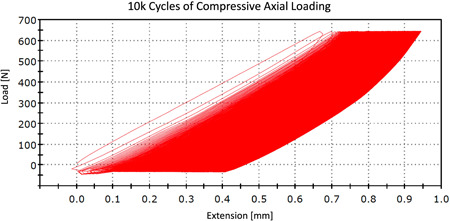
Hysteresis load‐displacement curve of a specimen subjected to 10,000 cycles of compressive axial loading. After 10,000 cycles, only small displacement of the actuator was noticed (0.4 mm) and no change of the shape of the hysteresis loop. This was consistent with the high stiffness and cyclic stability of tested constructs [Color figure can be viewed at wileyonlinelibrary.com]

The analysis of PIT measurements did not reveal statistically significant differences in PIT among the screws placed in different screw positions within the same treatment group as well as among the screws placed in the constructs assigned to different treatment groups (*p* > .05; Tables 1 and 2). The analysis of biomechanical testing revealed mean actuator displacement in G5K and G10K groups of 1.04 ± 0.2 and 1.07 ± 0.1 mm, respectively, and mean construct stiffness of 10,409.28 ± 1,888 and 9982.57 ± 1,394 N/mm^2^, respectively. The difference between G5K and G10K was not statistically significant (*p* = .211 and *p* = .449, respectively). The hysteresis load‐creep curve did not significantly change its shape during testing which confirmed the cyclic stability of tested constructs (Figure 4).

**Table 2:** Mean values and SD of PIT and PRT values (N‐cm) for proximal (1–4) and distal (5–8) combined screw positions

Screw position	Control		5,000 Cycles		10,000 Cycles	
	PIT, N‐cm	PRT, N‐cm	PIT, N‐cm	PRT, N‐cm	PIT, N‐cm	PRT, N‐cm
Combined prox (1–4)	420.5 ± 7.7	340.2 ± 47.6^a,α^	422.6 ± 9.1	299.5 ± 54.3^a,β^	421.3 ± 10.9	278.6 ± 71.1^a,β^
Combined dist (5–8)	421.3 ± 8.9	346.7 ± 49.8^a,α^	424.8 ± 11.6	307.0 ± 58.9^a,β^	422.4 ± 11.5	313.5 ± 64.3^a,β^
Average	420.9 ± 0.6	343.5 ± 4.6^α^	423.7 ± 1.5	303.3 ± 5.3^β^	421.9 ± 0.7	296.0 ± 24.6^β^

*Note*: a, Statistically significant difference between the screw positions within the same treatment groups were labeled using different alphabetic letters and α, statistically significant difference between the treatment groups for the same screw position were labeled with different Greek letters.

Abbreviations: PIT, peak insertion torque; PRT, peak reverse torque.

The detailed analysis of the PRT measurements from the individual screw positions revealed that screws placed in positions 2, 3, and 4 had the greatest PRT reduction after 5000 and 10,000 cycles (Table 1; Figure  3). The only significant reduction in PRT was for screws placed in position no. 3 and only after 10,000 cycles (*p* = .022).

**Figure 5:**
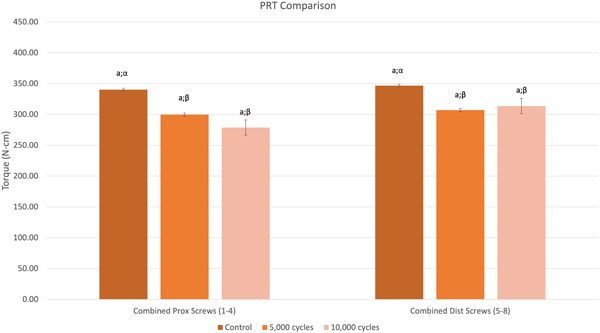
Combined PRT comparison between the treatment groups. Statistically significant difference in PRT between the screw positions within the same treatment groups are labeled using different alphabetic letters and statistically significant difference between the treatment groups for the same screw position are labeled with different Greek letters. PRT, peak reverse torque [Color figure can be viewed at wileyonlinelibrary.com]

The PRT reduction in the distal screws was less than in the proximal screws. The differences in PRT between individual screw positions were not statistically significant for C and G5K groups (*p* = .562 and *p* = .255, respectively; Table 1). In G10K, the screws placed in positions no. 5 exhibited significantly greater PRT as compared with the screws placed in positions no. 3 (*p* = .013; Table 1). Furthermore, the analysis of averaged PRT values from combined screws placed in the proximal positions from 1 to 4 (group 1) and in the distal positions from 5 to 8 (group 2) did not reveal statistically significant differences in PRT between groups 1 and 2 within the treatment groups (*p* > .05; Table 2; Figure 5).

## 4 DISCUSSION

PRT is a validated method in orthodontics to evaluate the strength of bone and dental implant interface.^10^, ^11^, ^12^, ^17^, ^18^, ^19^, ^20^ Recently, orthopedic studies found it to be valuable to assess primary stability of locking hole inserts placed in locking plates subjected to cyclic loading as well as to evaluate and compare osseointegration of non‐self‐tapping and self‐tapping screws placed in a dynamic compression plate.^18^, ^19^ Another study on dental implants used PRT to evaluate the primary stability of various lengths of dental implants subjected to cyclic loading.^27^ PRT measures the torque required to remove the implant from the tissue or orthopedic plate, thus indirectly assesses the stability of the screw and bone or screw and plate interface.^17^ The study utilized this method to assess the primary, mechanical stability of the locking‐head screws after their placement within the locking plate subjected to the compressive loading.

Based on the results and statistical analysis, the hypothesis that cyclic loading has a significant negative effect on the interface stability of screws placed in locking plate constructs used to bridge segmental bone defects was accepted. The screws placed distal to the osteotomy tended to experience a lesser reduction in PRT than screws placed proximally. This phenomenon could be explained with a non‐uniform distribution of loading across the locking plates resulting in the greater load applied to the proximal screws.^18^, ^28^ Locking plates have been described as single‐beam constructs that provide angle‐stable fixation.^4^, ^5^ All the locking‐head screws are placed perpendicularly to the surface of the plate and theoretically, there should be no motion between the single elements of the beam.^4^, ^5^ Bone loading is directly transmitted to the compression forces applied to the individual screws which are proportional to the amount of loading experienced by the bone segment.^4^, ^5^, ^6^, ^7^, ^8^ It can be therefore concluded that the screws placed in the proximal bone segment experienced more loading than the screws placed in the distal bone segment.

Interestingly, the screws placed further away proximally from the osteotomy (#1) experienced a lesser reduction in PRT than the screws placed more adjacent to it. This phenomenon was less pronounced for the distal screws possibly due to the greater bone mineral density (BMD) and thicker cortex of the distal tibia.^18^, ^28^ Although outside of the scope of these studies, this finding may have clinical relevance in cases where locking plates are applied in bones with low BMD, such as osteoporosis. The screw‐bone interface could be even more compromised due to the low quality of the surrounding bone.

Significant PRT reduction was observed after 5000 cycles and an additional, 5000 cycles did not significantly decrease the PRT. This observation implies that PRT reduction occurred within 5000 cycles and plateaued during the subsequent 5000 cycles. Future research could explore the effect of cyclic loading in the range from 0 to 5000 cycles and that of greater than 10,000 cycles. However, the focus of this study was to evaluate the effect of cyclic loading on the stability of the screws in the postoperative period and the number of cycles was chosen accordingly to the previous study to mimic the postoperative loading conditions in the most suitable way.^23^

Torque reduction measured immediately after placement of the screws documented the expected “time 0” difference between the insertion and reverse torque. This difference emphasizes the importance of careful screw insertion to optimize the stability of the screw and plate interfaces. The small standard deviation among PRT results within the control group confirmed the consistency of screws placement in this model and justified the use of a torque‐limiting screwdriver. The standard deviation became proportionally greater relative to the number of cycles applied to the constructs under consistent, controlled cyclic loading which confirms the significant effect of cyclic loading on screw stability. The absolute lengths of the screws varied between the implants, however, the screw and bone contact length (working length of the screw) was similar among the implants since the cutting‐flute of self‐tapping screws extended beyond the far cortex warranting the good screw purchase. The screw working length and not the screw absolute length has been associated with the mechanical stability of the implant.^29^ Of particular interest, monocortical locking‐head screws have been shown to have reduced implant stability as compared with bicortical screws due to reduced screw purchase and reduced screw working length.^30^ The monocortical locking‐head screws are commonly used in fracture repair, especially when the purchase of the far cortex is not possible because of the placement of an intramedullary implant. Reduced screw stability with monocortical placement likely increases compressive loading at the screw‐bone interface and may have even greater effect on the screws. The evaluation of constructs fixed with the monocortical screws was not within the scope of this study.

The model of the non‐load‐sharing fracture using a full‐thickness segmental tibial defect was designed based on the experience in our lab. The rationale behind the size of the defect gap was to create the possible shortest working length of the plate to reduce the local yield stress experienced by the plate and screws.^31^ Working length of the orthopedic plate has been defined as the distance between the proximal and distal screw in closest proximity to the osteotomy.^1^, ^31^, ^32^ It has been found that a longer working length in plates used to stabilize fracture gaps below 6 mm compared with the shorter working length leads to a significant reduction in local yield stress experienced by the plate and screws adjacent to the osteotomy.^31^ In contrast, in fracture gaps greater than 6 mm, shorter working length leads to a reduction in local yield stress experienced by the plate and adjacent screws.^31^ The local yield stress is not as relevant in this experiment because the constructs were not loaded beyond the yield point. Under clinical settings, however, minimalization of the local yield stress concentration around the plate and screws adjacent to the bone defect (positions no. 4 and 5) should be considered. In an attempt to mimic the in vivo clinical scenario, the locking plate‐screws constructs were applied to the bone as they would be under clinical settings. Regardless of the size of the fracture gap, increase in the working length has been associated with decreased construct rigidity.^1^, ^31^, ^32^ The working length of the locking plate constructs was chosen to be 10 mm for the proximal and distal segment of the bone to maintain sufficient bone fragment between the osteotomy and adjacent screws. This was also consistent with our experience using orthopedic models of segmental defects in goat tibia.

Primary implant stability has been strongly correlated with implant osseointegration. Osseointegration results from peri‐implant osteogenesis and is influenced by several factors including implant material, the status of adjacent bone, mechanical stability, and loading conditions applied to the implant.^33^, ^34^, ^35^, ^36^, ^37^, ^38^ It has been found that micromotion between the implant and bone around 30 µm has a positive influence on osseointegration, however excessive micromotion above 150 µm has a negative effect on this process.^39^, ^40^ Our study suggests that locking head screws used in non‐load‐sharing fractures may be subjected to torsional micromotion between the screws and plate as well as between the screws and bone which may lead to reduced primary stability of the implant and increasing the risk of screw displacement. Osseointegration begins as soon as 10–14 days after implantation.^41^ Our study found that a significant reduction in the stability of locking‐head screws occurs within 5000 cycles of loading. It represents the immediate postoperative days as Healthy working people may be expected to take an average of 8873 ± 2757 steps per day as compared with goats who would be expected to take an average of 5380 ± 3092 steps per day.^42^, ^43^

Limitations of this study include the ex vivo nature of the experiment. All factors which could have potentially influenced the results of the study were controlled throughout the experiment and no confounding effects were found. However, extrapolation of these results to in vivo conditions and clinical patients should be performed with caution until future research can be done. The micromotion of the screw implants was not directly evaluated in a quantitative manner. This can be done using linear or rotational variable differential transformers.^44^ In order to limit study variables, this experiment was limited to only one clinical scenario in which physiologically normal bones were used. Future studies could implement this model and introduce variables, such as using bones with variable or low BMD, osteoporotic bones, use of monocortical screws with or without intramedullary constructs, and testing to yield point at the end of different cyclical loading conditions. Although the source of the orthopedic implants was a manufacturing company marketing specifically to the veterinary market, those implants are composed of standard 316L stainless steel and manufactured using identical standard procedures for orthopedic implants and do not limit the goals of the study, which was the effect of cyclical loading on the integrity of the screw‐bone interface.

## 5 CONCLUSION

This study documented a negative effect of cyclic loading on the early post‐implantation stability of screws placed within a locking plate construct used to stabilize non‐load‐sharing fractures ex vivo. Reduced primary stability could further negatively affect the osseointegration of the entire construct and impair the healing process. The results of this study will serve as a reference point for further studies on the primary stability of locking screws used in locking plate constructs.

## CONFLICT OF INTERESTS

The authors declare that there are no conflict of interests.

## AUTHOR CONTRIBUTIONS

Remigiusz M. Grzeskowiak conducted internal fixation procedures, biomechanical testing, organized and analyzed data as well as wrote the manuscript. Laura R. Freeman assisted with the preparation of the specimens, internal fixation procedures, and biomechanical testing. David P. Harper supervised data collection and analysis as well as manuscript writing. David E. Anderson supervised data collection, analysis as well as manuscript writing. Pierre‐Yves Mulon mentored the project, performed internal fixation procedures, biomechanical testing as well as supervised data collection, analysis, and manuscript writing. All authors have read and approved the final submitted manuscript.
